# The Short-Term Impact of Sleeve Gastrectomy on Bone Health

**DOI:** 10.31138/mjr.030324.tst

**Published:** 2025-01-24

**Authors:** Adel Ibrahim Azzam, Saad Ghanem, Ahmed Elzayat

**Affiliations:** Rheumatology and Rehabilitation Department, Faculty of Medicine, Al-Azhar University, Cairo, Egypt

**Keywords:** obesity, bone loss, bone turnover, bone mineral density, bariatric surgery, gastric sleeve

## Abstract

**Objective:**

To study short-term impact of sleeve gastrectomy (SG) on bone outcomes such as bone metabolism, bone turnover, and bone mineral density (BMD).

**Methods:**

The study included 69 bariatric surgery-eligible patients (37 males and 32 females) ranging in age from 30 to 45 years. Dual-energy x-ray absorptiometry (DXA) scans were used to calculate areal BMD (g/cm2) and T scores in the total hip, femoral neck, and lumbar spine (L1-L4). Serum levels of C-terminal telopeptide (CTX), type 1 collagen propeptide (P1NP), parathyroid hormone (PTH), and 25-hydroxy vitamin D (25(OH)D) were measured. All patients received baseline and 12-month postoperative evaluations.

**Results:**

The study included 69 individuals, 37 (53.62%) male and 32 (46.38%) female, aged 36.86 ± 6.31, with morbid obesity (mean BMI 46.32 ± 4.21 kg/m2) who underwent SG. The mean levels of circulating calcium, 25(OH)D, and PTH did not change significantly after surgery (p > 0.05) and remained within normal ranges twelve months later. Following surgery, bone turnover markers increased significantly (p < 0.05), with CTX showing a higher overall percentage increase than P1NP. The total hip and femoral neck BMD decreased significantly after surgery, but there was no significant difference in lumbar spinal BMD before and after surgery.

**Conclusion:**

This study demonstrates that BMD decreases drastically after SG, and bone loss is more pronounced at the hip and femoral neck sites 12 months after surgery than at the lumber spine. Regular follow-ups, including BMD measurements, are recommended after bariatric surgery, at least once a year.

## INTRODUCTION

Obesity is a medical disorder and a potentially preventable public health concern that is rapidly growing.^1^ Bariatric surgery is an effective strategy for dealing with obesity and associated complications.^2^ Obesity has reached epidemic proportions, making bariatric surgery a fundamental therapy for obesity that is refractory to normal weight-reduction measures, particularly when considerable weight-reduction outcomes are required to reduce adiposity-related health issues. There are several currently accepted clinical guidelines for the use of bariatric surgery in obesity,^3–5^ and the progress of surgical upward trends over the last decade reveals similarities and differences in the overall number and types of surgical and endoluminal approaches.^6^

In terms of typical bariatric surgery advancements, sleeve gastrectomy (SG) is steadily increasing, while Roux-en-Y gastric bypass (RYGB) and laparoscopic adjustable gastric band (AGB) are decreasing.^4^ When compared to RYGB, laparoscopic SG has demonstrated excellent excess weight reduction with minimal morbidity.^7^ Postsurgical weight reduction has been linked to improved health in regards to diabetes mellitus, hypertension, cardiopulmonary issues, dyslipidaemia, vulnerability to malignancies, problems with mobility, gastroesophageal reflux (despite varying results), mental and behavioural health, improved quality of life, and a lower risk for cardiac fatal outcomes, cerebrovascular accidents, and overall mortality.^8^ SG, on the other hand, is more likely to exacerbate or induce gastroesophageal reflux than to improve it.^9^ Furthermore, there is currently rising concern in regards to the potential effect of bariatric surgery on the integrity of the bones, with evidence pointing to the development of increased bone loss and bone fragility following surgery.^10^

Our study aims to determine the short-term impact of SG on bone outcomes such as bone metabolism, bone turnover, and bone mineral density (BMD).

## MATERIAL AND METHODS

Between May 2021 and July 2023, we recruited 69 bariatric surgery-eligible patients (37 males and 32 females) aged 30 to 45 years from our university hospital's General Surgery department. Patients had to satisfy the traditional criteria for SG, comprising a BMI≥40 kg/m^2^ or a BMI≥35 kg/m2 in conjunction with high-risk concurrent disorders or lifestyle-limiting obesity-related physical problems. Following surgery, all patients received 1000–1200 mg of elemental calcium as well as 3000 IU of cholecalciferol per day.^11^ We excluded participants who were wheelchair-bound, pregnant, perimenopausal, had metabolic bone disease, such as osteoporosis, or were taking drugs with documented impacts on bone metabolism.

All patients had baseline and 12-month post-operative evaluations. Areal BMD (g/cm^2^) and T scores was evaluated at the total hip, femoral neck, and lumbar spine (L1–L4) using dual-energy x-ray absorptiometry (DXA) scans (Hologic, Waltham, MA, USA) prior to and 12 months following surgery. Serum C-terminal telopeptide (CTX) levels were determined using an enzyme-linked immunosorbent assay (ELISA). The serum type 1 collagen propeptide (P1NP) was evaluated using radioimmunoassay (RIA). Serum Parathyroid hormone (PTH) and 25-hydroxy vitamin D (25(OH)D) levels were determined using supersensitive electrochemiluminescence immunoassays (Roche Diagnostics, Mannheim, Germany). The serum level of calcium was determined using an automated biochemical analyser (Hitachi, Ltd., Tokyo, Japan).

All individuals provided written consent for participation in this study.

### Statistical analysis

The raw data was statistically analysed using Statistical software (version 6.0). The data is provided as means ± SEM. The 1-year changes in the examined variables across each group (1 year after surgery versus baseline) were compared using the paired Student t test for the dependent variables. P-values ≤0.05 were deemed to be statistically significant across all comparisons.

## RESULTS

The study comprised 69 individuals, 37 (53.62%) male and 32 (46.38%) female, aged 36.86 ± 6.31, with morbid obesity (mean BMI 46.32 ± 4.21 kg/m2) who underwent SG. **Table 1** shows our patients' baseline evaluations prior to SG.

**Table 1.** Baseline features of the study group prior to operation.

	**All study participants (n =69)**
Age (years)	36.86 ± 6.31
Weight (kg)	120.43 ± 13.23
BMI (kg/m^2^)	45.34 ± 4.13
Serum calcium (mg/dl)	9.66 ± 0.61
Serum 25(OH) D (ng/ml)	23.62 ± 7.83
Serum PTH (pg/ml)	52.22 ± 16.84
P1NP (ng/ml)	52.63 ± 22.74
CTX (ng/ml)	0.41 ± 0.1
**DXA scan**	
Spine aBMD (g/cm^2^)	0.97 ± 0.15
Spine T-Score	0.8 ± 1.1
Total Hip aBMD (g/cm^2^)	1.12 ± 0.13
Total Hip T-Score	0.91 ± 0.87
Femoral Neck aBMD (g/cm^2^)	0.98 ± 0.18
Femoral Neck T-Score	0.7 ± 1.12

PTH: parathyroid hormone; P1NP: type 1 collagen propeptide; CTX: The C-terminal telopeptide; DXA: dual-energy x-ray absorptiometry; aBMD: areal bone mineral density.

The mean value of circulating calcium, 25(OH)D, and PTH levels did not change considerably after surgery and were within normal ranges at twelve months after surgery. There was a considerable rise in bone turnover markers after surgery, with CTX demonstrating an even greater overall percentage increase than P1NP (**Table 2**).

**Table 2.** Effects of sleeve gastrectomy on body composition, bone metabolism, and turnover markers.

**Variable**	**Baseline**	**12 months**	**Delta 12 months % change**	***p*-value**
Weight (kg)	120.43 ± 13.23	65.63 ± 37.24	−29.95 ± 8.05	<0.05
BMI (kg/m^2^)	45.34 ± 4.13	32.22 ± 4.64	−28.97 ± 7.83	<0.05
Serum calcium (mg/dl)	9.66 ± 0.61	9.42 ± 0.34	−1.62 ± 4.32	>0.05
Serum 25(OH)D (ng/ml)	23.62 ± 7.83	27.34 ± 10.23	11.94 ± 33.45	>0.05
Serum PTH (pg/ml)	52.22 ± 16.84	51.42 ± 15.23	−3.34 ± 21.96	>0.05
P1NP (ng/ml)	52.63 ± 22.74	103.98 ± 31.43	114.25 ± 63.65	<0.05
CTX (ng/ml)	0.41 ± 0.1	0.97 ± 0.37	162.76 ± 41.68	<0.05

PTH: parathyroid hormone; P1NP: type 1 collagen propeptide; CTX: C-terminal telopeptide.

The BMD of the lumbar spine, total hip, and femoral neck decreased following the procedure compared to the baseline evaluation. The total hip and femoral neck BMD decreased considerably after surgery; however, there was no substantial difference in the lumbar spinal BMD prior to and following surgery (**Table 3**).

**Table 3.** Effects of sleeve gastrectomy on bone mineral density.

**Variable**	**Baseline**	**12 months**	**Delta 12 months % change**	***p*-value**
Spine T-Score	0.8 ± 1.1	0.5 ± 1.21	−0.3 ± 0.46	>0.05
Total Hip T-Score	0.91 ± 0.87	0.15 ± 0.63	−0.82 ± 0.28	<0.05
Femoral Neck Hip T-Score	0.7 ± 1.12	−0.54 ± 0.71	−0.97 ± 0.52	<0.05

## DISCUSSION

Morbid obesity is a condition with a significant energy excess. In the past few years, bariatric surgery has received more attention as an effective intervention. Nonetheless, the most popular forms of bariatric surgery, RYGB and SG, have been linked with postoperative issues caused by a lack of various essential elements and micronutrients.^12^

Individuals with obesity, even those who have not undergone bariatric surgery, might be at a higher likelihood of developing fragility fractures of the hip after they reach the age of sixty.^13^ This has been related to an inadequate level of vitamin D.^14^ Osteoporosis is frequently reported following bariatric surgery, particularly RYGB, and is thought to be directly related to a significantly reduced absorption of calcium and vitamin D.^15^ According to Saad et al.,16 bypassing the duodenum and proximal jejunum, which are important for calcium absorption, may lead to postoperative bone loss. However, other types of bariatric surgery, such as SG, has also been linked to decreased bone health and lower BMD.^17^

Our study found a decrease in BMD at the lumbar spine, total hip, and femoral neck 12 months after the SG operation compared to the baseline examination. SG resulted in a significant drop in the total hip and the femoral neck BMD but no significant change was noted in the lumbar spine BMD. These findings agreed with previous research.^18,19^

Since 1980, bariatric surgery has been consistently linked with an observed reduction in BMD.^20^ Several studies investigating the association between bariatric surgery, including RYGB or SG, and diminished bone mass have precisely revealed that substantial decreases in BMD develop during the first year following bariatric surgery.^21,22^

It has been reported that weight loss has been linked to significant decreases in BMD, especially in postmenopausal women and older men.^23,24^ Thus, following bariatric surgery, body weight is reduced by up to 30%, and this lower mechanical strain can lead to reduced bone formation, increased bone loss, and decreased BMD.^25^ Nevertheless, Ben-Porat et al.^26^ demonstrated that bone loss is mostly experienced during the time of rapid weight loss and continues even after reaching a stable weight, indicating that weight loss alone might not be entirely responsible for the detrimental impact of bariatric surgery on bone.

The skeletal system undergoes changes after weight loss as a result of a variety of factors and interactions, some of which have unknown consequences.^27^ Without a doubt, post-operative metabolic alterations have a greater and more rapid impact on bone loss than typical weight loss. Nonetheless, these rapid changes are accompanied by reduced food consumption, altered micronutrient absorption, and additional physiological alterations.^26^

According to previous research, mechanical unloading, decreased initial calcium and vitamin D absorption, and hormonal changes (both gonadal and gut hormones) are the main contributors to postoperative bone loss. Other factors, such as decreased lean mass and increased bone marrow fat, do play a role.^16,28^

It has been reported that mechanical loading can affect the size, mass, and biomechanical properties of human bones^29^ hence, mechanical strain on the bones and joints during motion and exercise preserves BMD.^30^ According to Li et al.^29^ mechanical loading changes cause osteocytes to remodel bone locally via the sclerostin pathway. Losing weight reduces skeletal mechanical loading, which can worsen bones in the absence of physical activity or exercise.^26^ Thus, differences in bone density reduction between the hip and lumbar spine may be attributed to weight loss-induced differential off-loading of these areas as well as the increased degenerative changes that may impact lumbar spinal BMD measurement.

The disruption of the synthesis of several gastrointestinal hormones that control appetite, including ghrelin, could also explain the drop in BMD following SG.^31^

Bariatric surgery can significantly alter body composition and nutritional elements, leading to changes in bone turnover markers.^32,33^ The most commonly used biomarkers for bone production and resorption are P1NP and CTX, with blood plasma levels of both indicators generally increasing with going on bone loss.^19,34^ In the current study, the bone turnover biomarkers increased significantly after 12 months of SG, which is consistent with Paccou et al.^35^ and Salman et al.36 who reported that these markers can be elevated from the first week and remain elevated for 7 years post-surgery. It has been reported that P1NP and CTX levels increase by 50%–300% one year after SG, which is compatible with our findings.^22^

Our findings demonstrated a considerable rise in CTX and P1NP following surgery, with CTX rising more than P1NP, indicating that bone resorption predominates bone formation, which results in a worsening of bone microarchitecture and an increase in fracture risk that is independent of BMD.^37^

Our study found that individuals who underwent SG had lower blood calcium and PTH levels but higher 25(OH) D levels in comparison to the baseline evaluation.

In the current study, the mean blood level of 25(OH) D at the initial evaluation was quite low (23.62 ± 7.83), which is consistent with other studies.^38,39^ Despite the fact that low vitamin D levels have been associated with malnutrition and limited exposure to sunshine as a result of decreased physical activity, some studies have demonstrated that there is enhanced uptake of vitamin D in fat cells, thereby lowering blood vitamin D levels.^40,41^

According to reports, many studies have found that bariatric surgery can exacerbate or even cause vitamin D deficiency, which can lead to bone loss,^42^ this problem is more common in malabsorptive procedures such as RYGP because they bypass the intestinal segment involved in vitamin D absorption, resulting in more severe vitamin D loss than restrictive techniques such as SG.^43,44^

Our study found that serum vitamin D levels increased non-significantly (27.34 ± 10.23) after SG. This mild increase could be attributed to adipose tissue loss following surgery, or could be caused by accelerated food passage, a topic that requires further investigation.^45^

Another plausible explanation for this mild rise in vitamin D could be the clear effect of postoperative vitamin D supplementation. Although there was no difference in blood 25(OH) D levels, BMD decreased significantly following SG. Hofsø et al.^22^ found that bone loss persists despite vitamin D supplementation, especially after RYGB. These findings could suggest that either losing weight or other variables, such as a lack of vitamin K or zinc, are contributing to bone deterioration.^46^

In accordance with Hamoui et al.,^47^ our study found no significant decrease in serum calcium and PTH levels after surgery. It is anticipated that levels of PTH would fall, as decreased body weight caused by dietary means lowers PTH levels in those with morbid obesity.^48^

The study's limitations include the short follow-up period, the lack of a control group, and the small sample size. Another limitation is that interpretations and generalizations of the results must be approached with caution because dietary patterns vary greatly around the world. Studies on the effects of bariatric surgery on other variables, particularly vitamin K status and its role in postoperative bone health, are still insufficient.

## CONCLUSION

This study confirms that bone loss is more noticeable at the hip and femoral neck sites 12 months after surgery than at the lumber spine, and that BMD decreases significantly after SG. All bariatric patients should receive regular follow-ups, including BMD measurements, bone metabolism indicators, and nutritional assessments. BMD should be measured at least once a year after bariatric surgery.

## ETHICS APPROVAL AND CONSENT TO PARTICIPATE

The ethical committee for the rheumatology department at Al-Azhar University's Faculty of Medicine granted approval to this study. It adheres to the rules outlined in the Helsinki Declaration. An informed consent form was created and signed by each participant.

## CONSENT FOR PUBLICATION

Not applicable.

## CONFLICT OF INTEREST

The authors declare no conflict of interest.

## FUNDING

For the purpose of conducting the research, writing, and/or publishing this paper, the authors received no funding.

## AVAILABILITY OF DATA AND MATERIAL

The information will be provided upon justifiable request.

## AUTHORS CONTRIBUTIONS

The analysis and work supervision were conceptualised and planned by A.I.A., while S.G. assisted with the drafting of the paper and helped interpret the findings. A.E. helped with the data gathering. The final version was read and approved by the authors.
